# Effects of β-alanine and L-histidine supplementation on carnosine contents in and quality and secondary structure of proteins in slow-growing Korat chicken meat

**DOI:** 10.1016/j.psj.2022.101776

**Published:** 2022-02-11

**Authors:** Chanadda Suwanvichanee, Panpradub Sinpru, Kasarat Promkhun, Satoshi Kubota, Cindy Riou, Wittawat Molee, Jirawat Yongsawatdigul, Kanjana Thumanu, Amonrat Molee

**Affiliations:** ⁎ School of Animal Technology and Innovation, Institute of Agricultural Technology, Suranaree University of Technology, Nakhon Ratchasima, 30000, Thailand; † School of Food Technology, Institute of Agricultural Technology, Suranaree University of Technology, Nakhon Ratchasima, 30000, Thailand; ‡ Synchrotron Light Research Institute (Public Organization), Nakhon Ratchasima, 30000, Thailand

**Keywords:** carnosine, β-Alanine, L-Histidine, slow-growing chicken, synchrotron radiation-based Fourier transform infrared microspectroscopy

## Abstract

Carnosine enrichment of slow-growing Korat chicken (**KRC**) meat helps differentiate KRC from mainstream chicken. We aimed to investigate the effects of β-alanine and L-histidine supplementation on the carnosine synthesis in and quality and secondary structure of proteins in slow-growing KRC meat. Four hundred 21-day-old female KRC were used, and a completely randomized design was applied. The chickens were divided into 4 experimental groups: basal diet (A), basal diet supplemented with 1.0% β-alanine (B), 0.5% L-histidine (C), and 1.0% β-alanine combined with 0.5% L-histidine (D). Each group consisted of 5 replicates (20 chickens per replicate). On d 70, 2 chickens per replicate were slaughtered, and the levels of carnosine, anserine, and thiobarbituric acid reactive substances were analyzed. Biochemical changes were monitored using synchrotron radiation-based Fourier transform infrared microspectroscopy; 5 chickens per replicate were slaughtered, and the meat quality was analyzed. Statistical analysis was performed using ANOVA and principal component analysis (**PCA**). Group D chickens exhibited the highest carnosine meat content, followed by those in groups B and C. However, amino acid supplementation did not affect anserine content and growth performance. Higher carnosine levels correlated with increasing pH_45 min_ and decreasing drip loss, cooking loss, shear force, and lipid oxidation. PCA revealed that supplementation with only β-alanine or L-histidine was related to increased content of β-sheets, β-turns, and aliphatic bending groups and decreased content of α-helix groups. This study is the first to report such findings in slow-growing chicken. Our findings suggest that KRC can synthesize the highest carnosine levels after both β-alanine and L-histidine supplementation. Higher carnosine contents do not adversely affect meat quality, improve meat texture, and alter the secondary structures of proteins. The molecular mechanism underlying carnosine synthesis in chickens needs further study to better understand and reveal markers that facilitate the development of nutrient selection programs.

## INTRODUCTION

Korat chicken (**KRC**) is a crossbreed between Thai indigenous Leung Hang Khao sires and Suranaree University of Technology synthetic breed dams. The main purpose of the crossbreeding was to provide an alternative breed to promote the occupation of smallholder farmers in Thailand and Southeast Asia. Korat chicken is categorized as a slow-growing chicken (Poompramun et al., 2021) because it has a lower growth rate than typical chicken. Its body weight reaches market weight (approximately 1.2 kg–1.3 kg) within 9 to 10 wk of age (Hang et al., 2018). The lower performance of KRC, particularly of female KRC, is a significant disadvantage for farmers.

Because KRC is a crossbred chicken, half of its genetic background comes from the Thai indigenous chicken. Hata et al. (2021) concluded that Thai and most other indigenous chickens in South-East Asia and southern China originate from the same ancestor and were also selected from a similar environment. Hence, KRC could be used as a model for slow-growing chickens.

Sex is an important factor influencing carnosine content in chicken meat (Intarapichet and Maikhunthod, 2005). Previous studies reported that female chicken meat accumulates higher carnosine content than male chicken meat (Intarapichet and Maikhunthod, 2005; Jung et al., 2013). Carnosine is a major histidine-containing dipeptide consisting of β-alanine and L-histidine (Barbaresi et al., 2019). Anserine, a derivative of carnosine, is composed of β-alanine and 3-methyl histidine (Drozak et al., 2013). Carnosine and anserine play a very important role in human health by protecting and relieving pain from diseases such as aging, cancer, Alzheimer's disease, Parkinson's disease, and the complications of type-2 diabetes (Hipkiss et al., 2013). Therefore, enriching carnosine in KRC is a good strategy to improve the competitiveness of smallholder farmers and differentiate KRC from commercial broilers.

Carnosine synthesis and its content in muscle can be enhanced when amino acid substrates are supplemented in the diet (Kopec et al., 2020; Kralik et al., 2015; Qi et al., 2018). Regarding growth performance, the role of carnosine and its substrate (β-alanine and L-histidine), including other related factors, has been investigated in fast-growing chickens. Van Vught et al. (2008) found that histidine promotes growth hormone secretion, whereas Bhattacharya et al. (2015) reported that β-alanine could enhance physical fitness, and Tiedje et al. (2010) reported that β-alanine could act as a neurotransmitter to regulate the secretion of hormones related to growth and development. In addition, there are many studies related to the effect of carnosine and its substrates on growth performance in fast-growing chicken, some of which found significant effects (Cong et al., 2017b; Qi et al., 2018; Kopec et al., 2020), but that of Hu et al. (2009) did not. Regarding meat quality, Kralik et al. (2015) reported that supplementation of a higher amount of β-alanine or L-histidine in the diet could increase carnosine content in chicken breast meat, but does not affect thiobarbituric acid reactive substances (**TBARS**). Cong et al. (2017a) found that chickens that were fed a carnosine-supplemented diet could produce high-quality meat by increasing pH_45 min_, drip loss, and cooking loss. Dietary β-alanine supplementation reduces the shear force of the meat in broiler chicks (Qi et al., 2018). Kralik et al. (2018) reported that β-alanine and L-histidine supplementation combined with magnesium oxide decreased pH_45 min_ and drip loss. Unfortunately, data on varying metabolism (Sirri et al., 2011), movement behavior (Castellini et al., 2016), and ability to resist oxidative stress (Mattioli et al., 2017) in slow-growing chickens are limited compared to those on fast-growing chickens. To date, the effect of substrates of carnosine synthesis and its content in the muscle on growth performance and meat quality in slow-growing chickens remains unclear.

Regarding meat quality changes, Beattie et al. (2004) suggested that shear force, tenderness, and beef texture are influenced by changes in biochemical compounds such as the ratio of α-helices to β-sheet and the hydrophobicity of the myofibrils in the environment. Katemala et al. (2021) demonstrated that the relative content of β-sheet is positively correlated with the shear force of KRC meat. Moreover, Fourier transform infrared (**FTIR**) spectrometry has been used to study meat quality in terms of biochemical changes (Bocker et al., 2007; Candoğan et al., 2020). Synchrotron radiation-based Fourier transform infrared (**SR-FTIR**) microspectroscopy is a powerful and sensitive approach for detecting the vibrations of molecules that provide information about protein secondary structure, lipids, and glycogen. This technique can be used to analyze samples at the micron level or micro-sample areas (Wang et al., 2015). Yu (2004) suggested that the SR-FTIR technique can be used for feed science and animal nutrition research. Therefore, the results using synchrotron-FTIR to measure the transformation of biochemical compounds in our study are expected to be more interesting than those using FTIR spectrometry.

The goal of this research was to increase carnosine synthesis in KRC using genetic manipulation. We aimed to elucidate the adverse effects of carnosine content on growth performance and meat quality. Hence, in this study, an experiment was designed using β-alanine and L-histidine supplementation (substrate of carnosine synthesis) to increase carnosine levels in KRC breast meat. Kralik et al. (2015) identified that supplementation with 1% β-alanine or 0.5% L-histidine significantly increased carnosine content in chicken breast meat (20.48 and 25.96%, respectively) but did not affect its lipid oxidation. Therefore, we investigated carnosine synthesis in slow-growing KRC that were fed 1% β-alanine and 0.5% L-histidine-supplemented diet and monitor the effect of supplementation on growth performance and meat quality, including biochemical compounds and secondary structure of proteins in meat using SR-FTIR microspectroscopy. To the best of our knowledge, this is the first study on the effect of dietary supplementation on biochemical and physiochemical changes in slow-growing chicken meat. Our findings will provide insights into carnosine synthesis and its effect on chicken meat properties before designing a breeding program in slow-growing chickens.

## MATERIALS AND METHODS

### Ethics Statement

The experiments were approved by the Ethics Committee on Animal Use of the Suranaree University of Technology, Nakhon Ratchasima, Thailand (document ID: U1-02631-2559).

### Experimental Design and Chicken Handling

Female KRC produced in the Suranaree University of Technology farm were used for the study. When the chickens were 21 days old, they were randomly assigned to 4 experimental diet groups using a completely randomized design with 5 replicates per group and 20 chickens per replicate. The mean and SD of the chicken body weight was approximately 266.04 ± 3.03 g.

The 4 experimental diets, all of which were formulated based on the National Research Council nutrient recommendations (1994), were as follows: basal diet (A), basal diet supplemented with 1.0% β-alanine (B), 0.5% L-histidine (C), and 1.0% β-alanine combined with 0.5% L-histidine (D). The ingredient and nutrient compositions of the experimental diet for growers (22–42 days) and finishers (43–70 days) are shown in Table 1, some of which have already been published by Kubota et al. (2021).

**Table 1:** Ingredient and nutrient composition of experimental diets in different growing phases.

	Grower (22 to 42 d)^1^				Finisher (43 to 70 d)^1^			
	A	B	C	D	A	B	C	D
Ingredients (kg)								
Corn	59.40	57.52	58.30	56.64	67.30	65.14	66.14	64.44
Soybean meal (44% CP)	30.80	28.40	28.22	28.78	26.30	26.70	26.50	26.70
Full fat soybean	2.42	5.50	5.80	5.20	0.00	0.00	0.00	0.00
Rice bran oil	4.00	4.20	3.80	4.50	3.04	3.80	3.50	4.00
L-Lysine	0.18	0.18	0.18	0.18	0.19	0.19	0.19	0.19
DL-Methionine	0.21	0.21	0.21	0.21	0.14	0.14	0.14	0.14
Salt	0.35	0.35	0.35	0.35	0.35	0.35	0.35	0.35
Calcium carbonate	1.42	1.42	1.42	1.42	1.20	1.20	1.20	1.20
MDCP (P21)	1.02	1.02	1.02	1.02	1.28	1.28	1.28	1.28
Premix^2^	0.20	0.20	0.20	0.20	0.20	0.20	0.20	0.20
β-Alanine	0	1.00	0	1.00	0	1.00	0	1.00
L-Histidine	0	0	0.50	0.50	0	0	0.50	0.50
Total (kg)	100	100	100	100	100	100	100	100
Calculated composition								
ME (kcal/kg)	3,113	3,116	3,112	3,111	3,112	3,116	3,118	3,110
Crude protein, %	19.39	19.30	19.39	19.29	17.08	17.10	17.08	17.04
Crude fiber, %	3.60	3.56	3.58	3.55	3.32	3.30	3.31	3.29
Ether extract, %	6.94	7.60	7.29	7.82	5.81	6.49	6.23	6.66
Calcium, %	0.90	0.90	0.90	0.90	0.86	0.86	0.86	0.86
Total phosphorus, %	0.57	0.57	0.57	0.56	0.60	0.59	0.59	0.59
Analytical value of L-Histidine (%)	0.40	0.44	0.97	1.02	0.30	0.30	0.93	0.97

1 Treatment groups are A (control), B (supplementation with 1.0% β-alanine), C (supplementation with 0.5% L-histidine), and D (supplementation with 1.0% β-alanine and 0.5% L-histidine), respectively.

2 Premix (0.5%) provided the following per kilogram of diet: 15,000 IU of vitamin A, 3,000 IU of vitamin D3, 25 IU of vitamin E, 5 mg of vitamin K3, 2 mg of vitamin B1, 7 mg of vitamin B2, 4 mg of vitamin B6, 25 ug of vitamin B12, 11.04 mg of pantothenic acid, 35 mg of nicotinic acid; 1 mg of folic acid, 15 µg of biotin, 250 mg of choline chloride, 1.6 mg of Cu, 60 mg of Mn, 45 mg of Zn, 80 mg of Fe, 0.4 mg of I and 0.15 mg of Se.

The chickens were raised in an open house, and the stocking density was 8 birds/m^2^. Food and water were provided ad libitum. The vaccination program was performed as per guidelines set by the Department of Livestock Development, Bangkok, Thailand.

The chickens were weighed, feed intake data were collected weekly, and data were used to calculate the body weight gain, average daily feed intake, average daily gain, and feed conversion ratio at 21, 42, and 70 d.

### Sample Collection

On d 70, 10 chickens per group (2 chickens/ replicate) were randomly tagged and stunned by chloroform. Then chickens were slaughtered by decapitation and bled. Approximately 10 g of breast meat (*M. pectoralis major*) was packed in a vacuum bag and frozen at −80°C. Samples were analyzed for carnosine, anserine, and TBARS. The other portion (10 g) was kept in a zip-lock bag, stored at 4°C for 24 h, and used for biochemical analysis.

Five chickens per replicate were randomly tagged and fasted for 24 h. They were then stunned by electric shock, decapitated, bled, scalded before mechanical de-feathering, and manually eviscerated. Carcasses were chilled at 4°C for 24 h, and then the breast meat was removed for meat quality measurement.

### Meat Quality Measurement

*pH.* The breast meat pH (25 samples/treatment) was measured using an electronic pH meter (UltraBasic pH meter, Model UB10, Denver Instrument, Bohemia, NY) at 45 min and 24 h postmortem. The pH was measured 3 times in the same area, and the probe was washed with ultrapure water between different sample measurements.
*Drip loss.* At 24 h postmortem, drip loss was calculated using the method establish by Kralik et al. (2018). The breast meat was cut in 2 × 3 cm pieces with an approximate weight of 7 g, mopped, and weighed before being kept in a plastic bag at 4°C for 24 h. Then, the sample was mopped and reweighed to calculate drip loss using the following equation:DripLoss(%)=[(initialweight−finalweight)/initialweight]×100%
*Cooking loss.* Cooking loss was determined using the method described by Kim et al. (2016) with slight modifications. The breast meat was cut in pieces of approximately 2 × 3 × 1 cm^3^ from the same location and weighed. Samples were cooked in a water bath at 80°C until the core temperature reached 71°C. The samples were then cooled at room temperature for 3 h and weighed. The percentage of cooking loss was calculated using the following equation: Cooking loss (%) = [(initial weight-cooked weight)/initial weight] × 100%.
*Shear force.* The shear force of cooked samples (2 × 1 × 1 cm^3^) was determined following the method of Kim et al. (2016) using a Texture Analyzer (TA-XT Plus, Stable Micro System Ltd., Surrey, UK). A Warner-Bratzler shear attachment was used at a test speed of 2 mm/s. The samples were cross-sectionally cut into muscle fibers. The shear force values of 25 samples per treatment were recorded.

### TBARS

Breast meat stored at −80°C for 5 mo was used for TBARS measurements according to the method described by Kong et al. (2008) with slight modifications. A ground sample (1 g) was homogenized with 3 mL 7.5% trichloroacetic acid (w/v). The homogenate was centrifuged at 10,000 × *g* and 4°C for 10 min. One milliliter of the supernatant was added to 1 mL of 0.02 M thiobarbituric acid, and incubated at 95°C for 20 min. Subsequently, the sample was cooled at 5°C for 5 min, and the absorbance was measured at 532 nm using a BioTek Epoch Microplate Spectrophotometer (Epoch, BioTek, VT). Malonaldehyde bis (diethyl acetal) (ACROS Organics, Gothenburg, Sweden) was used as a standard.

### Carnosine and Anserine Measurements

The carnosine and anserine contents were determined using the method of Mora et al. (2007) with slight modifications. Breast meat (0.1 g) was homogenized with 900 µL of 0.01 N HCl for 2 min and centrifuged at 12,000 × *g* at 4°C for 10 min. Supernatants were filtered through a 0.45-μm syringe filter. The filtrate (250 µL) was mixed with 750 µL of acetonitrile and stored at 4°C for 20 min. Then, the sample was centrifuged at 10,000 rpm at 4°C for 10 min, filtered through a 0.2-μm syringe filter, and stored at −20°C until use. Carnosine and anserine were separated on an Atlantis HILIC silica column (4.6 × 150 mm, 3 µm, Waters Corporation, Milford, MA) equipped with high-performance liquid chromatography (HPLC 1260, Agilent Technology, Santa Clara, CA). Mobile phase A containing 0.65 mM ammonium acetate in 75% acetonitrile at pH 5.5 and mobile phase B containing 4.55 mM ammonium acetate in 30% acetonitrile at pH 5.5 were used. The separation conditions were determined by the linear gradient of phase B from 0 to 100% for 13 min at 1 mL/min. Twenty microliters of the sample obtained after the addition of acetonitrile were injected. Dipeptides were detected at a wavelength of 210 nm. Quantification was performed using the external standard carnosine and anserine (Sigma-Aldrich, St. Louis, MO) at 25°C.

### SR-FTIR Microspectroscopy

After chilling, 10 breast meat samples from each group were cross-sectionally cut into a muscle fiber size of 1 × 1 cm and placed into an aluminum foil block. The frozen tissues were cut using a cryostat (Leica CM1950, Leica Biosystems Nussloch GmbH, Nussloch, Germany) at a thickness of 7 µm and placed on the IR window (Crystran Ltd, Dorset, UK), then placed in a vacuum desiccator for 2 to 3 d before the SR-FTIR measurement.

Two muscle sections of each chicken were subjected to spectra analysis. FTIR spectra were measured at BL4.1 Infrared spectroscopy & Imaging, Synchrotron Light Research Institute, using the SR-FTIR spectrometer with a synchrotron light source in the mid-IR region. Spectra were collected on a Bruker FTIR spectrometer (Vertex70, Bruker Optics, Ettlingen, Germany) coupled to a Bruker Hyperion 2000-IR Microscope (Bruker Optik GmbH, Ettlingen, Germany) with a 36x objective, coupled to an MCT detector cooled with liquid nitrogen covering a measurement range from 4,000 to 800 cm^−1^. The FTIR spectra were obtained in the transmission mode, collecting 64 scans with a 10 × 10 µm aperture size at a resolution of 6 cm^−1^ over a measurement range from 4,000 to 800 cm^−1^. Each group comprising 400 spectra (20 spectra × 2 muscles replicates × 10 chickens) were processed using the OPUS 7.5 software (Bruker Optics Ltd.).

The integral areas were determined using second-derivative processing at the spectral regions from 3,000 to 900 cm^−1^, including lipid, amide I, amide II, CH-binding of lipid, amide III, and glycogen.

Curve fitting of amide I was determined using the original spectra after preprocessing to calculate the integral areas of amide I (1,700 to 1,600 cm^−1^), α-helix (1,644, 1,655 cm^−1^), β-sheet (1,630 cm^−1^), β-turn (1,670 cm^−1^), and antiparallel (1,689 cm^−1^) regions based on Gaussian and Lorentzian functions.

### Statistical Analysis

#### Principal Component Analysis

Principal component analysis (**PCA**) was used to identify the biochemicals in the spectral ranges from 3,000 to 2,800 cm^−1^ and 1,800 to 900 cm^−1^. All spectral data were preprocessed using the Savitzky-Golay algorithm for second derivative transformations at 13 smoothing points and normalized with extended multiplicative signal correction using the Unscrambler X Multivariate Data Analysis software (version 10.1, Camo Analytics, Oslo, Norway). The 400 spectra were averaged into 20 spectra per treatment, and outliers were removed until 5 spectra per treatment for groups or clusters were obtained using PCA. The bi-plot correlation was used to represent the clustered differentiation of data, and related variables were recalculated using the two-dimensional scatter plot of PCA with the predominant spectral range. The high loading SR-FTIR spectra were selected for multivariate analysis with dipeptide content and physicochemical properties. Data for all variables were weighted using an SD weighting process, and the relationship between variables was investigated using PCA bi-plot correlation.

#### Significant Difference Analysis

ANOVA was used to analyze the effect of experimental diets on growth performance, meat quality, TBARS, dipeptide content, the ratio of integral area, and secondary structure ratio. Significant differences between the means of the treatments were determined using Tukey's multiple tests. A *P*-value of < 0.05 was considered statistically significant. SPSS Version 16.0 for Windows (SPSS Inc., Chicago, IL) was used for statistical analysis.

## RESULTS AND DISCUSSION

### Carnosine and Anserine Contents in KRC Breast Meat

The carnosine and anserine contents in KRC breast meat are presented in Table 2. The carnosine content was the lowest in the control group. Chickens fed a diet supplemented with β-alanine (B), L-histidine (C), or both amino acids (D) showed higher carnosine contents (*P* < 0.05). The highest level of carnosine was found in group D, with a 52.8% increase compared to that of the control group. However, amino acid supplementation had no significant effect on the anserine content (*P* > 0.05).

**Table 2:** Carnosine and anserine contents in Korat chicken breast meat.

Parameter	Treatment group^1^				SEM	*P*-value
	A	B	C	D		
Carnosine (µg/g)	2,756.6^c^	3,484.6^b^	3,659.8^b^	4,212.5^a^	82.88	<0.001
Anserine (µg/g)	10,577.2	10,391.6	10,312.7	10,272.8	282.47	0.88

Results were averaged from 10 chickens per treatment.

1 Treatment groups are A (control), B (supplementation with 1.0% β-alanine), C (supplementation with 0.5% L-histidine), and D (supplementation with 1.0% β-alanine and 0.5% L-histidine), respectively.

a-c Mean values with different superscripts in the same row indicate significantly different at *P*-value < 0.05.

The results revealed that KRC could synthesize carnosine at approximately 2.76 mg/g. Kojima et al. (2014) reported that the carnosine content in the breast meat of 79-wk-old female Black Bond Silky Fowls was approximately 7.98 mg/g. Jung et al. (2013) reported that the carnosine content in the 20-wk-old breast meat from 5 lines of female Korean native chicken was approximately 1.69 to 1.83 mg/g, whereas Khumpeerawat et al. (2021) reported that the carnosine contents in the meat of 84-day-old black Chinese and KU Phupan chickens were approximately 5.01, and 5.27 mg/g, respectively. Fortunately, the carnosine level that KRC can synthesize is in the middle range of slow-growing chickens.

Carnosine synthesis can be improved when the chickens are fed carnosine synthase substrates. The carnosine content in the meat of chickens in groups B and C, supplemented with only β-alanine or L-histidine, respectively, was higher than that in the control group (26.42 and 32.76%, respectively). Regarding β-alanine supplementation, our results align with many previous studies (Tomonaga et al., 2006; Kralik et al., 2015). As demonstrated by Qi et al. (2018), β-alanine promotes the expression of carnosine-related transporters, and carnosine synthase increases the carnosine content. Carnosine synthesis based on the combined use of supplemented L-histidine and available β-alanine in the blood and muscle (Kai et al., 2015) can explain the observed increase in carnosine content when the diet was only supplemented with L-histidine. Moreover, the synthesis of carnosine may be one of the mechanisms by which animals try to control the L-histidine content balance in body.

The highest carnosine content was found when the diet was supplemented with both amino acids, consistent with the hypothesis that both amino acids are limiting amino acids for carnosine synthesis (Kai et al., 2015; Qi et al., 2018). This result, however, contrasted with that of Kopec et al. (2020), showing that the carnosine content did not differ compared to the results obtained using only β-alanine or L-histidine. Two possible reasons may explain these contrasting results. First, Barbaresi et al. (2019) reported that in fast-growing broilers, the available L-histidine is primarily directed towards muscle protein synthesis, but in slow-growing chickens, the synthesis of anserine and carnosine may have been favored by higher availability of L-histidine. Second, slow-growing chickens may have a better ability to store carnosine in the muscle. Conversely, slow-growing chickens are resistant to oxidative stress, as confirmed by Lengkidworraphiphat et al. (2020).

When compared with the other groups, a significant difference in the anserine content was not found in the L-histidine supplementation group or the combined amino acid supplementation group. These results may be explained by the fact that anserine can be synthesized via 2 pathways: carnosine methylation (Boldyrev and Severin, 1990) or 3-methyl histidine and β-alanine synthesis (Drozak et al., 2013). Methionine is converted to S-adenosylmethionine that is a common co-substrate for supplying methyl group in metabolic process (Ramadan et al., 2021). Therefore, it is possible that when methionine is scarce, it is prioritized for protein synthesis before being used for other functions, including the transfer to methyl group and binding with L-histidine to generate anserine.

### Carnosine Content on Growth Performance

KRC performance was expected to improve when the diet was supplemented with carnosine synthase substrates; however, contrary to our expectation, the results showed no significant difference in performance, including feed intake, body weight gain, average daily feed intake, average daily gain, body weight, and feed conversion ratio, among the different groups in each growth phase (*P* > 0.05; Table 3).

**Table 3:** Effect of amino acid supplementation on the growth performance of Korat chickens.

Parameter	Treatment group^1^				SEM	*P*-value
	A	B	C	D		
22‒42 d						
FI (g)	1,018.64	1,092.50	1,037.32	1,038.99	18.67	0.07
BWG (g)	381.97	386.21	382.48	390.17	5.68	0.72
ADFI (g)	48.51	52.02	49.40	49.47	0.89	0.07
ADG (g)	18.19	18.39	18.21	18.58	0.27	0.72
BW 42 d (g)	635.97	643.21	637.98	644.17	6.10	0.74
FCR	2.67	2.83	2.71	2.66	0.05	0.15
43‒70 d						
FI (g)	1,960.78	1,940.13	1,964.78	1,933.38	59.71	0.98
BWG (g)	512.64	501.04	523.21	562.64	24.05	0.33
ADFI (g)	70.03	69.29	70.17	69.05	2.13	0.98
ADG (g)	18.31	17.90	18.69	20.09	0.89	0.33
BW 70 d (g)	1,148.61	1,144.25	1,161.19	1,206.81	26.01	0.34
FCR	3.85	3.91	3.77	3.46	0.19	0.38
22‒70 d						
FI (g)	2,979.42	3,032.63	3,002.10	2,972.37	68.99	0.93
BWG (g)	894.61	887.25	910.69	952.81	25.65	0.30
ADFI (g)	60.80	61.89	61.27	60.66	1.41	0.93
ADG (g)	18.26	18.11	18.48	19.45	0.52	0.30
FCR	3.34	3.43	3.32	3.13	0.12	0.36

Abbreviations: ADFI, average daily feed intake; ADG, average daily gain; BW, body weight; BWG, body weight gain; FCR, feed conversion ratio;FI, feed intake.

Results were averaged from 25 chickens per replicate, 100 chickens per treatment.

1 Treatment groups are A (control), B (supplementation with 1.0% β-alanine), C (supplementation with 0.5% L-histidine), and D (supplementation with 1.0% β-alanine and 0.5% L-histidine), respectively.

It has been reported that oxidative stress affects poultry production (Surai and Fisinin, 2016). Oxidative stress occurs when there is an imbalance between reactive oxygen species (**ROS**) levels and antioxidant activity related to environmental factors (sunlight, thermal irradiation, air temperature, humidity, and movement) and animal's characteristics (species, gender, and rate of metabolism). Increase in ROS can decrease productive performance (Surai et al., 2019). This is in agreement with the study by Fouad et al. (2016), who reported that the increase of ROS leads to lipid peroxidation of intestine and pancreas cell walls, negatively affecting nutrient digestion and absorption. Lin et al. (2004) reported that the oxidative stress-induced effect of corticosterone in chicken is related to a reduction in body weight and poorer feed efficiency. However, slow-growing chickens can tolerate stress (Mattioli et al., 2017), and these chickens may not require carnosine, which acts as an antioxidant. For this reason, we could not detect significant differences in performance.

### Carnosine Content on Meat Quality and Lipid Oxidation

Amino acid supplementation did not affect pH_24 h_ (*P* > 0.05), whereas but led to an increase in pH_45 min_, the ability to retain water in meat and a decrease in the shear force (*P* < 0.05). The results, shown in Table 4, demonstrated that chickens fed an amino acid-supplemented diet (groups B, C, and D) tended to produce superior meat quality compared to that of the control group.

**Table 4:** Effect of amino acid supplementation on Korat chicken meat quality.

Parameter	Treatment group^1^				SEM	*P*-value
	A	B	C	D		
pH_45min_	5.27^b^	5.41^a^	5.33^ab^	5.40^a^	0.03	0.01
pH_24h_	5.36	5.39	5.39	5.38	0.02	0.759
Drip loss (%)	13.64^a^	13.17^ab^	11.11^b^	12.61^ab^	0.53	0.02
Cooking loss (%)	25.83^a^	24.55^b^	24.55^b^	24.05^b^	0.27	0.002
Shear force (kg)	3.31^a^	3.50^a^	2.94^b^	2.81^b^	0.08	<0.001

Results were averaged from 25 chickens per treatment.

1 Treatment groups are A (control), B (supplementation with 1.0% β-alanine), C (supplementation with 0.5% L-histidine), and D (supplementation with 1.0% β-alanine and 0.5% L-histidine), respectively.

a-c Mean values with different superscripts in the same row indicate significantly different at *P*-value < 0.05.

The superior meat quality may be due to a reduction in the decline in postmortem pH (pH_45 min_). When an animal dies, carnosine removes lactic acid and regulates Ca^2+^ discharge from the sarcoplasmic reticulum, reducing ATP consumption during postmortem glycolytic metabolism (Culbertson et al., 2010). As carnosine acts as a proton-sequestering molecule produced by anaerobic glycolysis (Boldyrev et al., 2013), it leads to a gradual decline in pH_45 min_ with an increase in the water holding capacity of the final meat product, affecting its functional properties (Kim et al., 2016). Moreover, carnosine can prevent oxidative protein modification by combining with reactive carbonyl species to inhibit protein carbonylation (Hipkiss et al., 2001). As a result, the meat from the amino acid-supplemented group had lower drip loss, shear force, and cooking loss.

**Figure 1:**
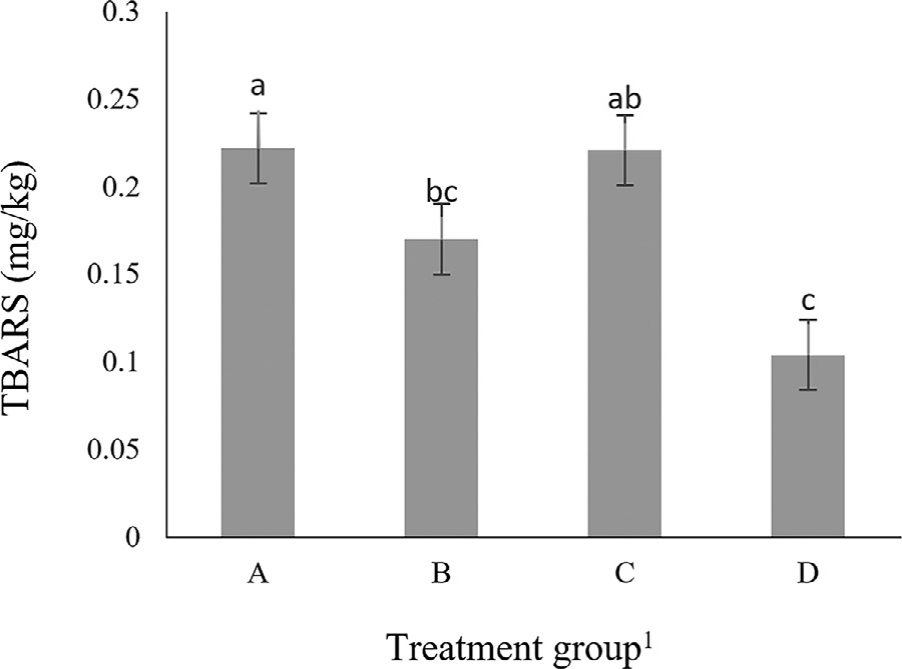
TBARS value in Korat chicken breast meat of the different experimental groups. Results were averaged from 10 chickens per treatment. Abbreviation: TBARS, thiobarbituric acid reactive substances.^1^Treatment groups are A (control), B (supplementation with 1.0% β-alanine), C (supplementation with 0.5% L-histidine), and D (supplementation with 1.0% β-alanine and 0.5% L-histidine), respectively.

TBARS (mg MDA/kg of tissue) was the lowest in group D (*P* < 0.05, Figure 1), demonstrating the anti-oxidant characteristics of carnosine. Metal ions catalyze the formation of advanced lipid oxidation end-products (Negre-Salvayre et al., 2008). The high level of carnosine in meat acts as a strong inhibitor of lipid oxidation end-products (Boldyrev et al., 2013), leading to lower lipid oxidation in broilers (Cong et al., 2017b).

### SR-FTIR Application for Determining the Intensity Ratios of Biomolecules and Secondary Structure Proteins From Different Carnosine Contents

**Figure 2:**
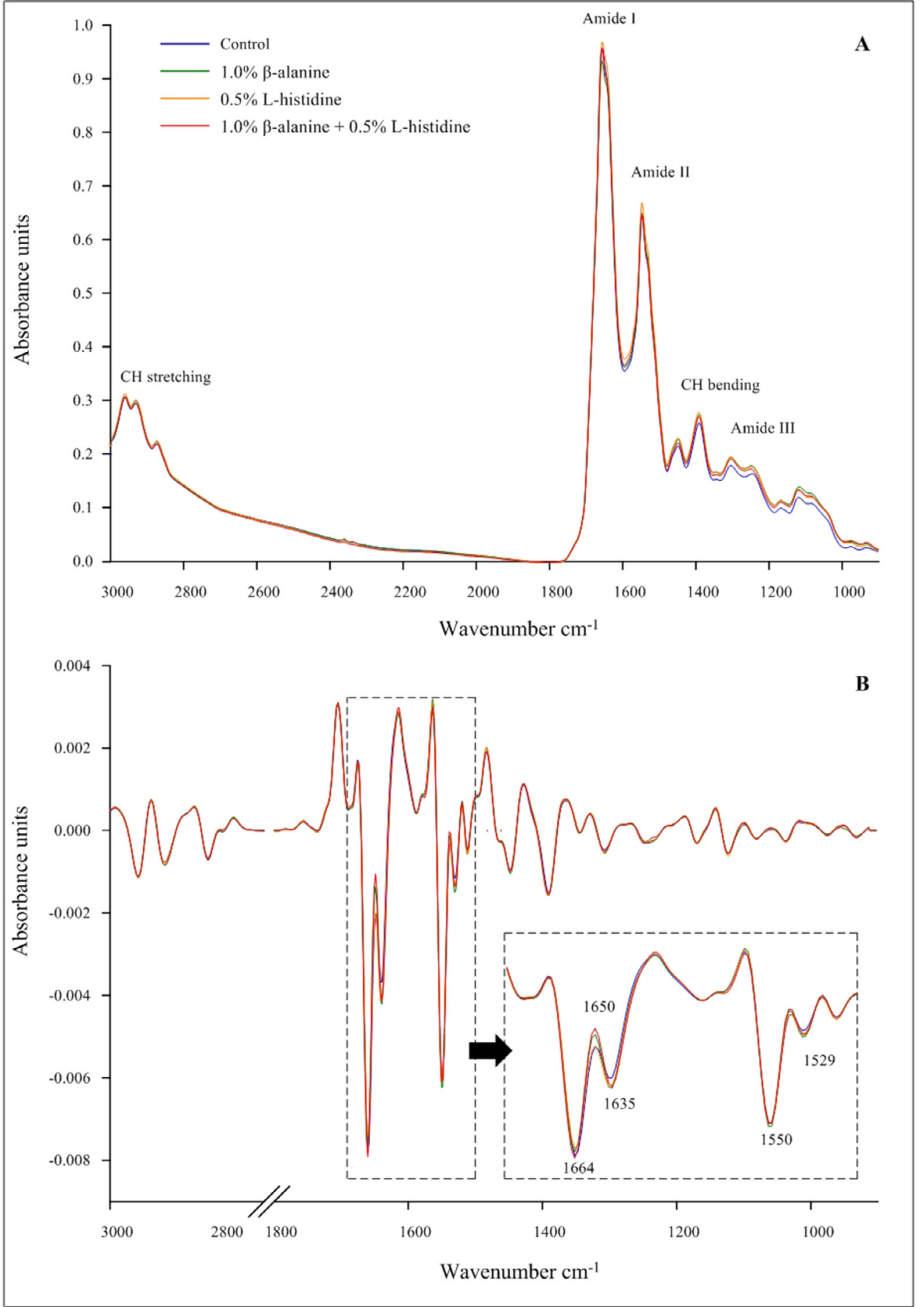
Average synchrotron radiation-infrared (SR-IR) spectra of the original spectra (A) and second derivative spectra (B) in the fingerprint region of the wave number at 3,000 to 900 cm−^1^ in Korat chicken breast meat of the different experimental groups.

The average original and second derivative spectra in the fingerprint region of the wave number at 3,000 to 900 cm^−1^ of KRC breast meat from the different amino acid supplementation groups are shown in Figures 2A and B, respectively. The average second derivative spectra from the 4 treatments clearly separated the peak high and peak ratios at 1,664 cm^−1^, 1,650 cm^−1^, and 1,635 cm^−1^ representing amide I and 1,587 cm^−1^, 1,550 cm^−1^, and 1,529 cm^−1^ representing amide II, respectively.

The percentages of integration of each biomolecule in Table 5 revealed significant differences between amides I and II (*P* < 0.05). The integral area of amide I in group D was lower than that in the control group. In contrast, those of groups B and C were not significantly different compared with that of the control or group D. Regarding amide II, the integral areas of the control, B, and D groups were higher than that of group C (*P* < 0.05). In addition to lipid, amide III, CH bending, and glycogen/carbohydrate showed nonsignificant differences (*P* > 0.05). These results suggested that the supplemented amino acids strongly affected the change in the secondary structure of muscle proteins.

**Table 5:** The ratio of the integral area of biomolecules in Korat chicken breast meat determined using synchrotron radiation-Fourier transform infrared (SR-FTIR) microspectroscopy.

Biomolecule (wavenumber)	% Integral area				SEM	*P*-value
	A	B	C	D		
Lipid (3,000–2,800 cm^−1^)	10.49	10.69	11.04	10.67	0.29	0.610
Amide I (1,700–1,600 cm^−1^)	42.01^a^	39.52^ab^	40.03^ab^	38.43^b^	0.73	0.023
Amide II (1,600–1,550 cm^−1^)	29.68^ab^	32.62^a^	28.87^b^	32.57^a^	0.75	0.004
CH bending (1,450–1390 cm^−1^)	5.54	5.81	6.79	5.95	0.41	0.198
Amide III (1,320-1,220 cm^−1^)	3.38	3.91	3.99	3.61	0.28	0.421
Glycogen/Carbohydrate (1,200-900 cm^−1^)	7.98	6.92	8.12	8.23	0.35	0.065

Results were averaged from 400 spectra per treatment.

Treatment groups are A (control), B (supplementation with 1.0% β-alanine), C (supplementation with 0.5% L-histidine), and D (supplementation with 1.0% β-alanine and 0.5% L-histidine), respectively.

a-b Mean values with different superscripts in the same row indicate significantly different at *P*-value < 0.05.

The secondary structure of the protein in the amide I region is composed of β-sheet, α-helix, β-turn, and antiparallel, and their integral areas are presented in Table 6. Significant differences in the secondary structures of amide I were detected. When the control group was used as the reference point, the relative β-sheet content in group D was lower than that of the control group, whereas the lowest relative β-turn content was found in the control group. These results confirm the results for amide I, shown in Table 5.

**Table 6:** Ratio of secondary structures in Korat chicken breast meat determined using synchrotron radiation-Fourier transform infrared (SR-FTIR) microspectroscopy.

Trait	% Curve fitting				SEM	*P*-value
	A	B	C	D		
β-sheet (1,630 cm^−1^)	22.24^a^	19.85^b^	20.71^ab^	18.72^b^	0.536	0.002
α-helix (1,644 cm^−1^, 1,655 cm^−1^)	49.44^ab^	49.59^ab^	47.85^b^	50.74^a^	0.596	0.028
β-turn (1,670 cm^−1^)	15.61^c^	19.09^a^	18.14^ab^	17.63^b^	0.244	0.000
Antiparallel (1,689 cm^−1^)	12.70^b^	11.68^b^	14.50^a^	12.92^b^	0.384	0.001

Results were averaged from 400 spectra per treatment.

Treatment groups are A (control), B (supplementation with 1.0% β-alanine), C (supplementation with 0.5% L-histidine), and D (supplementation with 1.0% β-alanine and 0.5% L-histidine), respectively.

a-c Mean values with different superscripts in the same row indicate significantly different at *P*-value < 0.05.

**Figure 3:**
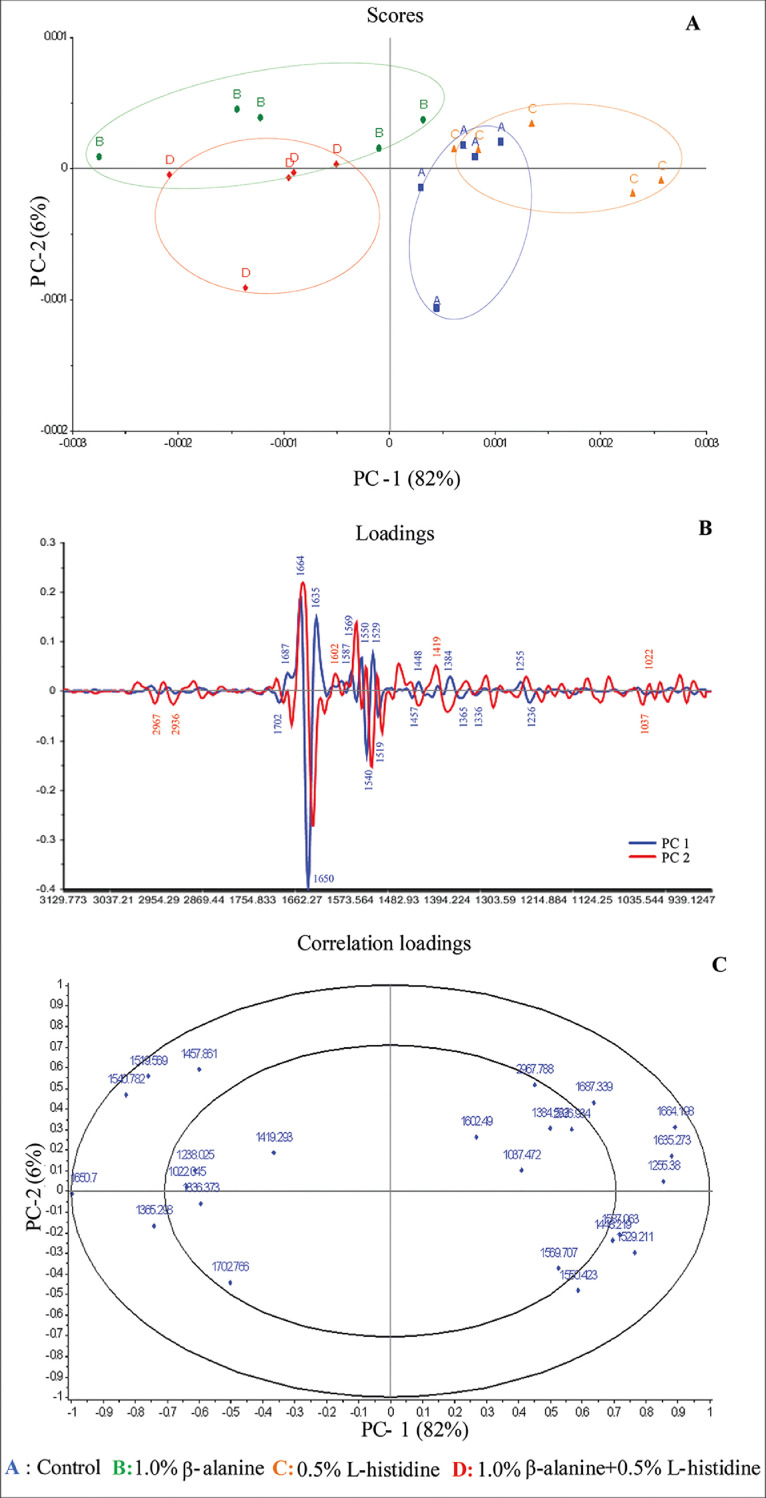
PCA score plot (A) for PC1 versus PC2 for 4 different experimental data, loading plot (B) detail of the effects of biomolecules on the meat of the different experimental groups, and correlation loading plot (C) for PC1 versus PC2 for biomolecules, at 88% total variance of Korat chicken breast meat of the different experimental groups. Spectra were collected (400 spectra per treatment) using second derivative processing at the spectral regions from 3,000 to 900 cm−^1^ by the outer and inner ellipse representing 100% and 50% of the variance, respectively. Abbreviation: PCA, principal component analysis.

The PCA score plot of the chicken meat biomolecule spectra is shown in Figure 3A, demonstrating that chicken meat was separated into 4 groups according to the experimental groups. The combination of PC1 and PC2 at 88% of the total variance could explain this finding Figures 3B and 3C show the details of the biomolecules that differentiated the meat in different groups. When the findings shown in Figures 3A and 3C were considered together, it was apparent that groups B and D were positively correlated with β-sheet (wavenumber 1,687 cm^−1^, 1,635 cm^−1^, 1,529 cm^−1^, and 1,255 cm^−1^), β-turn (wavenumber 1,664 cm^−1^), doublet due to the 2 protonated tautomers of histidine (1,587 cm^−1^), amide II (1,550 cm^−1^), and CH and CH_2_ aliphatic bending (wavenumber 1,448 cm^−1^) groups. Groups A and C were positively correlated with α-helix (wavenumber 1,650 cm^−1^ and 1,540 cm^−1^), tyrosine (wavenumber 1,519 cm^−1^), and CH and CH_2_ aliphatic bending groups (wavenumber 1,457 cm^−1^ and 1,365 cm^−1^). The reference SR-FTIR bands with high correlation loading in KRC breast meat are shown in Table 7.

**Table 7:** The related Fourier transform infrared (FTIR) band assignment observed spectra in Korat chicken breast meat.

Wavenumber (cm^−1^) in literature	Wavenumber (cm^−1^) in our study	Definition of the spectral assignments*	References
2,957–2,953	2,960‒2,874	CH_3_ asymmetric stretching Lipids (mainly), proteins	Candoğan et al. (2020)
1,700‒1,715	1,702	C= O stretching Fatty acid	Mecozzi et al. (2012)
1,682 1,659 1,653 1,639	1,687 1,664 1,650 1,635	Amide I of β-sheet Amide I of β-turn Amide I of α-helix Amide I of β-sheet	Bocker et al. (2007)
1,575,1,594	1,587	Doublet due to the 2 protonated tautomers of histidine	Barth (2007)
1,567 1,556 1,544 1,527	1,569 1,550 1,540 1,529	Amide II of β-sheet Amide II Amide II of α-helix Amide II of β-sheet	Bocker et al. (2007)
1,518	1,519	Tyrosine	De Meutter and Goormaghtigh (2021)
1,460‒1,350	1,457‒1,365	CH and CH_2_ aliphatic bending group	Mecozzi and Sturchio (2017)
1,256 1,237	1,255 1,238	Amide III of β-sheet	Singh et al. (1993)
1,035	1,037	C-O, C-C str., C-O-H, C-O-C def. (of carbohydrates)	Lazar et al. (2014)
1,020‒1,022	1,022	Glycogen	

⁎ Resolution of 6 cm^−1^.

As demonstrated by these results, the carnosine content can affect the quality of the meat related to its texture and water retention ability, which is consistent with previous studies (Cong et al., 2017a; Kralik et al., 2018; Qi et al., 2018). However, this study is the first to use SR-FTIR to monitor biomolecules in meat with different carnosine contents, and the SR-FTIR results showed that different carnosine contents could change the relative contents of some biomolecules in meat. Correlation loading analysis using the physicochemical characteristics, integral area of the biomolecule, integral area of the secondary structure of proteins, and wave number from Figure 3C was performed to make these data more informative, and the results are discussed in the next subtopic.

### Correlation Loadings Plot of PCA Between SR-FTIR Spectra and Physicochemical Results From Different Carnosine Contents

**Figure 4:**
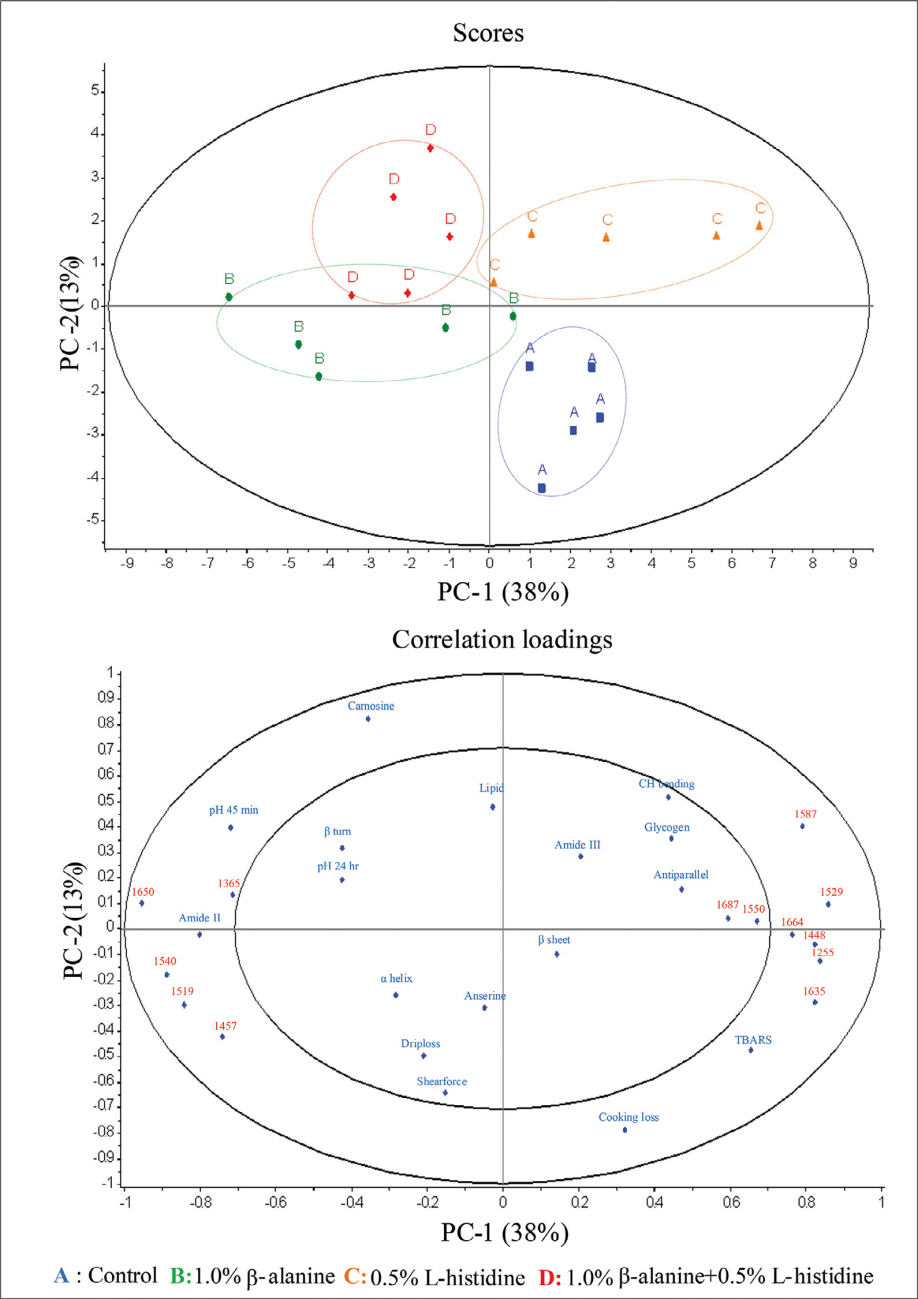
PCA score plot (upper) for PC1 versus PC2 for the 4 different experimental data and correlation loading plot (lower) for PC1 versus PC2 for physicochemical properties, biomolecules, secondary structure protein at 88% total variance in Korat chicken breast meat of the different experimental groups by the outer and inner ellipse representing 100% and 50% of the variance, respectively. Abbreviation: PCA, principal component analysis.

The score plot and correlation loading that expresses the relationship between SR-FTIR spectra and physicochemical results are shown in Figure 4 (upper and lower parts, respectively). This relationship explained approximately 51% of the total variation. All variables located in the outer circle region (carnosine, pH_45 min_, amide II, cooking loss, TBARS, and wavenumber 1,664 cm^−1^, 1,650 cm^−1^, 1,635 cm^−1^, 1,587 cm^−1^, 1,540 cm^−1^, 1,529 cm^−1^, 1,519 cm^−1^, 1,457 cm^−1^, 1,448 cm^−1^, 1,365 cm^−1^, and 1,255 cm^−1^) showed a significant correlation with the 4 experimental groups with a variance greater than 50%. Group A (Figure 4, upper part) was positively correlated with TBARS, cooking loss, α-helix (wave number 1,540 cm^−1^), tyrosine (wave number 1,519 cm^−1^), and CH and CH_2_ aliphatic bending groups (wave number 1,457 cm^−1^) (Figure 4, lower part). In group B, a positive correlation was found with amide II, β-sheet (wave numbers 1,635 cm^−1^, 1,529 cm^−1^, and 1,255 cm^−1^), β-turn (wave number 1,664 cm^−1^), and CH and CH_2_ aliphatic bending groups (wave number 1,448 cm^−1^). Group C was positively correlated with α-helix (wave number 1,650 cm^−1^) and CH and CH_2_ aliphatic bending groups (wave number 1,365 cm^−1^). In group D, a positive correlation was found with the carnosine content, pH_45 min_, and doublet due to the 2 protonated histidine tautomers (wave number 1,587 cm^−1^).

From the results, 2 interesting points need to be discussed. The first point is that carnosine levels can affect the physiological properties of meat by changing biomolecules in meat, as demonstrated using SR-FTIR. The second point is that β-alanine or L-histidine supplementation may decrease meat quality because it affects the increase in cooking loss and levels of TBARS, as demonstrated by the results, which aligned with those shown in Table 4 and Figure 1, respectively.

Supplementation with both amino acids (group D) can increase pH_45 min_, leading to a better maintenance of pH_45 min_. Simultaneously, the TBARS value decreased, resulting in an improved water-holding ability of muscle fibers. Interestingly, supplementation with both amino acids also increased the integral area to 1,587 cm^−1^, representing the binding of carnosine to Cu^2+^ (Torreggiani et al., 2000). This form of carnosine can act as a chelator ion, improving the antioxidant capacity (Güner and Alpsoy, 2015). Cong et al. (2017a) reported that increased antioxidant levels could improve the ability to maintain pH_45 min,_ decrease drip loss, cooking loss, and shear force. The results suggest that supplementation improves meat quality by changing the level of biomolecules in meat, as demonstrated by the SR-FTIR results. The effect of carnosine on anti-oxidant capacity needs to be investigated in future studies to confirm this.

Supplementation with either β-alanine or L-histidine has adverse effects on the physiological properties of meat, such as an increase in cooking loss and TBARS. The results may be caused by the increase in β-sheet, β-turn, and aliphatic bending groups. Residual amino acids from carnosine synthesis, free amino acids, might induce the oxidative modification of proteins (Zhang et al., 2013). Protein oxidation causes the unfolding of its secondary structure, which is transformed into β-sheets, β-turns (Li et al., 2020), and aliphatic bending groups (Herrero, 2008). Katemala et al. (2021) reported that the β-sheet relative content positively correlated with the shear force of KRC, and the result was confirmed by Beattie et al. (2004). Moreover, Kubota et al. (2021) found that the upregulated genes *LOC107051274, ACSBG1*, and *CAPNS2* and downregulated genes *MYO7B, MYBPH, SERPINH1*, and *PGAM1* may be related to meat tenderness in Korat chicken. However, it is still unclear how these genes change the protein secondary structure in this study. Further studies are required to clarify the molecular mechanism involved in meat quality.

This study confirmed that the carnosine content could be increased by amino acid supplementation (substrates of carnosine synthesis) in slow-growing KRC meat. Supplementation cannot improve the performance of chickens because of the ability of carnosine to resist oxidative stress. Furthermore, the highest carnosine synthesis was observed with supplementation of both β-alanine and L-histidine. Moreover, the water retention ability of muscle cells and pH_45 min_ can be improved. In addition, the results suggest that single β-alanine or L-histidine supplementation may negatively affect physiological properties, confirmed by the increase in β-sheet, β-turn, and aliphatic bending groups. However, we used only one level of both amino acids. Further studies are required to examine the optimum level of supplemented amino acids and genetics involved with carnosine synthesis.
